# Lack of causal association between heart failure and osteoporosis: a Mendelian randomization study

**DOI:** 10.1186/s12920-022-01385-8

**Published:** 2022-11-04

**Authors:** Heng Chen, Runze Ye, Xiaogang Guo

**Affiliations:** 1 Department of Cardiology, The First Affiliated Hospital, College of Medicine, Zhejiang University, Hangzhou, China; 2 Department of Cardiology, The First Affiliated Hospital, Zhejiang University School of Medicine, 79 Qingchun Road, Hangzhou, 310003 Zhejiang Province China

**Keywords:** Mendelian randomization, Causal association, Heart failure, Osteoporosis, Bone mineral density, Fracture

## Abstract

**Objectives:**

Heart failure (HF) has been implicated in osteoporosis. However, causality remains unestablished. Here, we sought to assess causal associations of genetic liability to HF with osteoporosis using Mendelian randomization (MR) analyses.

**Methods:**

Independent single nucleotide polymorphisms associated with HF at genome-wide significance were derived from a large genome-wide association study (GWAS) (including up to 977,323 individuals). We obtained summary statistics for forearm (FA) bone mineral density (BMD) (n = 8,143), femoral neck (FN) BMD (n = 32,735), lumbar spine (LS) BMD (n = 28,498), heel (HE) BMD (n = 426,824), and fracture (n = 1,214,434) from other GWAS meta-analyses. Inverse variance weighted (IVW) and several supplementary methods were performed to calculate the MR estimates.

**Results:**

Genetically determined HF has no causal effect on FA-BMD (odds ratio (OR) 1.17; 95% confidence interval (CI) 0.82, 1.66; *P* = 0.389), FN-BMD (OR 1.01; 95% CI 0.85, 1.19; *P* = 0.936), LS-BMD (OR 0.96; 95% CI 0.80, 1.17; *P* = 0.705), HE-BMD (OR 1.01; 95% CI 0.90, 1.13; *P* = 0.884), and fracture risk (OR 1.00; 95% CI 0.92, 1.10; *P* = 0.927). Complementary analyses returned broadly consistent results.

**Conclusion:**

This MR study provides genetic evidence that HF may not lead to an increased risk of reduced BMDs or fracture.

**Supplementary Information:**

The online version contains supplementary material available at 10.1186/s12920-022-01385-8.

## Introduction

Osteoporosis describes a chronic skeletal condition characterized by decreased bone mineral density (BMD), microarchitecture impairment, and increased fracture risk [1]. The forearm (FA), femoral neck (FN), lumbar spine (LS), and heel (HE) are the most common skeletal sites of osteoporosis [2]. BMD is highly heritable and polygenic [3, 4]; both ultrasound and dual-energy X-ray absorptiometry (DXA)-measured BMD have been applied to predict fracture [5, 6]. As a growing public health problem and its most critical complication, hip fracture, osteoporosis has been linked to high mortality and disability rate worldwide [7]. Nowadays, the disorder is largely preventable due to a better understanding of its risk factors, including low body mass index, estrogen deficiency, and smoking [1].

Heart failure (HF) is a common health threat that affects approximately 5.7 million Americans [8]. Patients with HF typically suffer from decreased cardiac function and disturbed neurohumoral status [9]. In recent years, HF has been recognized as a multisystem disorder associated with numerous metabolic disorders [10]. In the clinical setting, disturbed bone metabolism was found to be highly prevalent in adults with HF. Observational studies found evidence that HF was associated with reduced BMD in both sexes [11–13]. In addition, it was reported that lower BMD might be determined by HF severity (higher New York Heart Association class and N-terminal pro-B-type natriuretic peptide levels) [12, 14]. Cohort studies further revealed that patients with HF experienced an increased fracture risk [15, 16]. However, given that observational studies are easily biased by residual confounding, misclassification, and reverse causality [17], it remains an unanswered question whether HF has a causal effect on reduced BMD and fracture, or if it is just an episodic phenomenon when some shared risk factors linked both syndromes.

Mendelian randomization (MR) is commonly used to estimate the causal effect of exposures on outcomes where single nucleotide polymorphisms (SNPs) are utilized as instrumental variables (IVs) [18, 19]. According to Mendel's law of inheritance, the genetic variants are randomly assorted, independently of the environment and remain constant after conception. Those who inherit the allele were actually assigned to a higher specific trait [18, 19]. Therefore, the approach is considered a natural genetic counterpart of randomized controlled trials that can vastly diminish the influence of residual confounders and reverse causality. In this study, we conducted a two-sample MR study and sought to illustrate the causal associations of HF with reduced BMDs and fracture risk. We present the following article in accordance with the Strengthening the Reporting of Observational Studies in Epidemiology Using Mendelian Randomization (STROBE-MR) statement (Additional file 1: Table S1) [20].

## Methods

### Study design

**Fig. 1:**
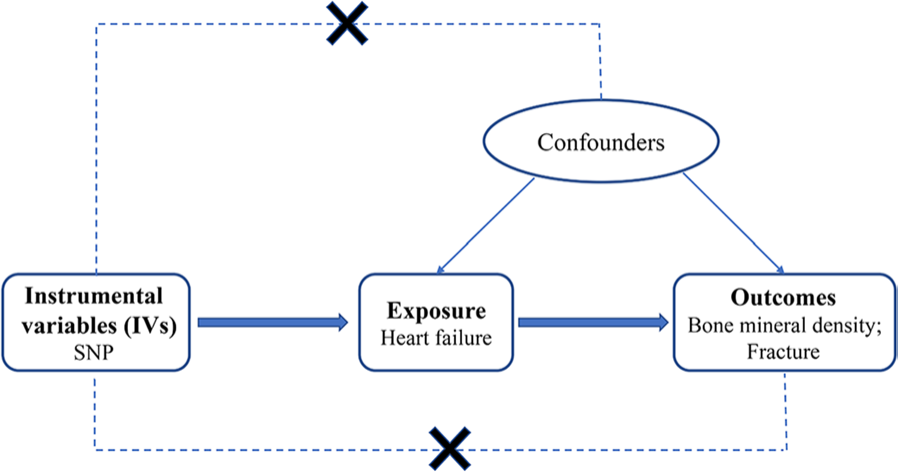
Schematic diagram showing the assumptions of Mendelian randomization analysis. Broken lines represent the horizontal pleiotropy that would violate the MR assumptions

There are three key(critical) assumptions for the MR approach. First, the IVs should be associated with the exposure in a significant way. Second, the IVs are not linked to potential confounders that may affect the exposure and (or) outcomes. Third, the IVs affect the outcomes exclusively by exposure but not via other pathways. (Fig. 1) [21]

Publicly available genome-wide association study (GWAS) summary statistics were selected for the analysis. No specific ethical approval or written informed consent was necessary.

### Data sources and IVs selection

Summary statistics for associations between genetic variants and FA-BMD, FN-BMD, and LS-BMD were obtained from a GWAS meta-analysis conducted by the Genetic Factors for Osteoporosis (GEFOS) consortium, in which BMD was measured by DXA-scanning [22] (Table 1). For HE-BMD estimated from ultrasound (n = 426,824) and fracture (n = 1,214,434), summary-level data were derived from another GWAS meta-analysis consisting of the UK Biobank and 23andMe cohort [23] (Table 1). Heel quantitative ultrasound can estimate BMD to the same extent as DXA-scanning, with the advantages of being mobile, inexpensive, and radiation-free [24]. Fracture cases were defined by two methods: 1) Hospital Episodes Statistics and 2) questionnaire-based self-reported fractures within the past five years [23].

**Table 1:** Detailed information of studies and datasets used for analyses

Phenotype	Data source	Sample size	Adjustment	Population
Heart Failure [25]	HERMES consortium	977,323	Principal components in individual studies	European
FA-BMD [22]	GEFOS consortium	8143	Sex, age, age^2^, weight	European
FN-BMD [22]	GEFOS consortium	32,735	Sex, age, age^2^, weight	
LS-BMD [22]	GEFOS consortium	28,498	Sex, age, age^2^, weight	
HE-BMD [23]	UK Biobank	426,824	Age, sex, genotyping array, assessment center and ancestry informative principal components 1 to 20	European
Fracture [23]	UK Biobank and 23andMe cohorts	1,214,434	Age, sex, genotyping array, assessment center and ancestry informative principal components 1 to 20	European

FA-BMD, forearm bone mineral density; FN-BMD, femoral neck bone mineral density; LS-BMD, lumbar spine bone mineral density; HE-BMD, heel bone mineral density; HERMES, Heart Failure Molecular Epidemiology for Therapeutic Targets; GEFOS, Genetic Factors for Osteoporosis

From the GWAS meta-analysis conducted by the Heart Failure Molecular Epidemiology for Therapeutic Targets (HERMES) Consortium, we obtained the summary statistics for HF (Table 1), where cases were diagnosed by clinical diagnosis of any etiology, with any etiology based on left ventricular ejection fraction [25]. Initially, the GWAS meta-analysis provided 12 SNPs strongly associated with HF at genome-wide significance (*P* < 5 × 10^−8^) [25]. We next performed a strict clumping procedure (r^2^ < 0.001 within a 10,000 kb window) referring to the European 1000 genomes project, with one SNP (rs140570886) excluded from the study. Detailed information on the 11 independent SNPs leveraged as IVs is provided in Additional file 1: Table S2. Next, we calculated the F statistic using the formula F = R^2^(n − 2)/ (1 − R^2^) to detect any weak IVs bias [26]. Here n represents the sample size; R^2^ refers to the proportion of variance explained by the selected SNPs and was calculated using the method described previously [27]. Altogether, they explained 1.42% of the phenotypic variability of HF (Additional file 1: Table S2). Besides, we did not find evidence of weak IVs bias since all these SNPs have F statistics higher than 10 (Additional file 1: Table S2). IVs absent in the outcome datasets were replaced with proxies in linkage disequilibrium (r^2^ > 0.8) if available. For those SNPs not available in the outcome datasets, we found proxies to replace them by searching a publicly available online tool [28]. Here, we found rs2395655, rs10738607, rs2519093, and rs11065979 to replace rs4135240, rs1556516, rs600038, and rs4766578, respectively. However, no eligible proxies were found for rs11745324, rs17617337, rs4746140, and rs55730499 (Additional file 1: Table S2).

### Statistical analyses

The MR estimates were calculated by combining the SNP-exposure, and SNP-outcome associations with inverse variance-weighted (IVW) used as the main method. The IVW approach can return an unbiased causal estimate when there is no horizontal pleiotropy or heterogeneity [29]. Several complementary analyses were used to test the robustness of the results: (1) Weighted median method. This method calculates the median of the instrumental variable estimates, providing unbiased results even if up to 50% of the IVs were invalid [30]; (2) Simple median method. It can be recognized as a weighted median estimator with equal weights [30]. (3) MR-Egger regression method [31]. This method provided a consistent ME estimate when the instrumental variables exhibited a pleiotropic effect. However, the result may be easily affected by outlier SNPs. (4) MR-Pleiotropy RESidual Sum and Outlier (MR-PRESSO) method. This method can identify and correct for pleiotropic outliers that may bias the results.

In the sensitivity analyses, we assessed heterogeneity among the SNPs by calculating the I^2^ statistic [32]. I^2^ statistics above 25% will be considered significantly heterogeneous [32], and then a multiplicative random effects IVW (mre-IVW) method was used [33]. Horizontal pleiotropy refers to IVs affecting outcomes through pathways other than the selected exposure. We here performed the MR-Egger regression and MR-PRESSO analyses to assess horizontal pleiotropy. Additionally, leave-one-out analyses were carried out to identify whether MR estimates were biased by any single SNP.

A Bonferroni-corrected *P* value of < 0.01 (0.05/5 outcomes) was considered significant. We performed the statistical analyses using TwoSampleMR [34] together with MR-PRESSO [35] packages in R software (version 4.1.0).

## Results

**Fig. 2:**
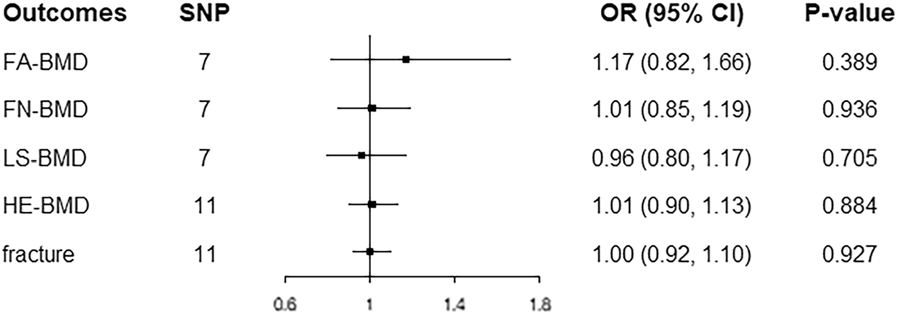
Mendelian randomization estimates of the causal associations of HF with bone mineral density and fracture risk. CI, confidence interval; FA-BMD, forearm bone mineral density; FN-BMD, femoral neck bone mineral density; HE-BMD, heel bone mineral density; LS-BMD, lumbar spine bone mineral density; OR, odds ratio; SNP, single-nucleotide polymorphism

As shown in Table 1, there was no sample overlap between Data sources for HF and BMDs and fracture. In analyses for the associations of HF with BMD at four skeletal sites, mre-IVW approach returned that HF was not causally associated with FA-BMD (odds ratio (OR) 1.17; 95% confidence interval (CI) 0.82, 1.66; *P* = 0.389), FN-BMD (OR 1.01; 95% CI 0.85, 1.19; *P* = 0.936), LS-BMD (OR 0.96; 95% CI 0.80, 1.17; *P* = 0.705), and HE-BMD (OR 1.01; 95% CI 0.90, 1.13; *P* = 0.884) (Fig. 2). In addition, mre-IVW analysis showed null causal association between HF and fracture (OR 1.00; 95% CI 0.92, 1.10; *P* = 0.927) (Fig. 2). Complementary analyses including weighted median, simple median, and MR-Egger regression methods yielded similar results, except for a suggestive association between HF and FN-BMD in the MR-Egger method (OR 1.71; 95% CI 1.18, 2.47; *P* = 0.037; Table 2).

**Table 2:** Complementary analyses of the associations of genetically predicted heart failure with bone mineral density and fracture

Outcome	SNPs, n	Method	OR	95% CI	*P* value
FA-BMD	7	Weighted median	1.13	0.80, 1.59	0.486
	7	Simple median	1.19	0.82, 1.71	0.362
	7	MR Egger	1.08	0.30, 3.97	0.909
	7	MR-PRESSO	NA	NA	NA
FN-BMD	7	Weighted median	1.03	0.89, 1.19	0.726
	7	Simple median	0.99	0.84, 1.17	0.932
	7	MR Egger	1.71	1.18, 2.47	0.037
	7	MR-PRESSO	NA	NA	NA
LS-BMD	7	Weighted median	1.02	0.85, 1.22	0.817
	7	Simple median	0.99	0.82, 1.21	0.941
	7	MR Egger	0.97	0.48, 1.95	0.927
	7	MR-PRESSO	NA	NA	NA
HE-BMD	11	Weighted median	1.03	0.99, 1.06	0.129
	11	Simple median	1.03	0.99,1.07	0.107
	11	MR Egger	0.90	0.58, 1.38	0.631
	11	MR-PRESSO^a^	1.02	0.99, 1.05	0.177
Fracture	11	Weighted median	1.00	0.89, 1.12	0.990
	11	Simple median	1.02	0.90,1.16	0.733
	11	MR Egger	1.00	0.71, 1.4	0.996
	11	MR-PRESSO	NA	NA	NA

FA-BMD, forearm bone mineral density; FN-BMD, femoral neck bone mineral density; LS-BMD, lumbar spine bone mineral density; HE-BMD, heel bone mineral density; SNP, single-nucleotide polymorphism; OR, odds ratio; CI, confidence interval; MR-PRESSO, MR-pleiotropy residual sum and outlier; NA, not applicable

^a^Outliers detected: rs4135240, rs56094641, rs56094641, and rs660240

**Fig. 3:**
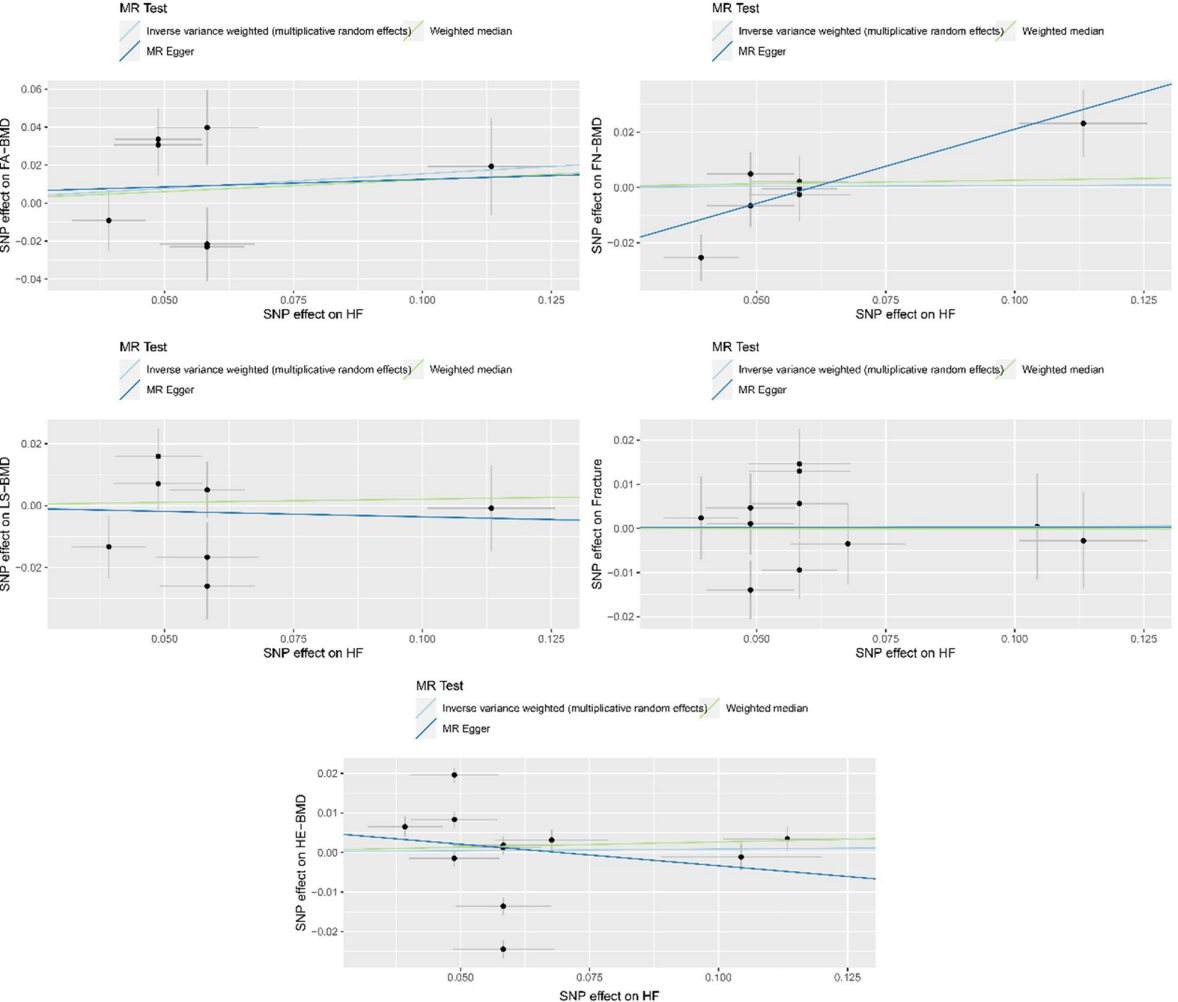
Scatter plots of the MR estimates for the association of HF with FA-BMD, FN-BMD, LS-BMD, HE-BMD, and fracture. HF, heart failure; FA-BMD, forearm bone mineral density; FN-BMD, femoral neck bone mineral density; LS-BMD, lumbar spine bone mineral density; HE-BMD, heel bone mineral density

**Fig. 4:**
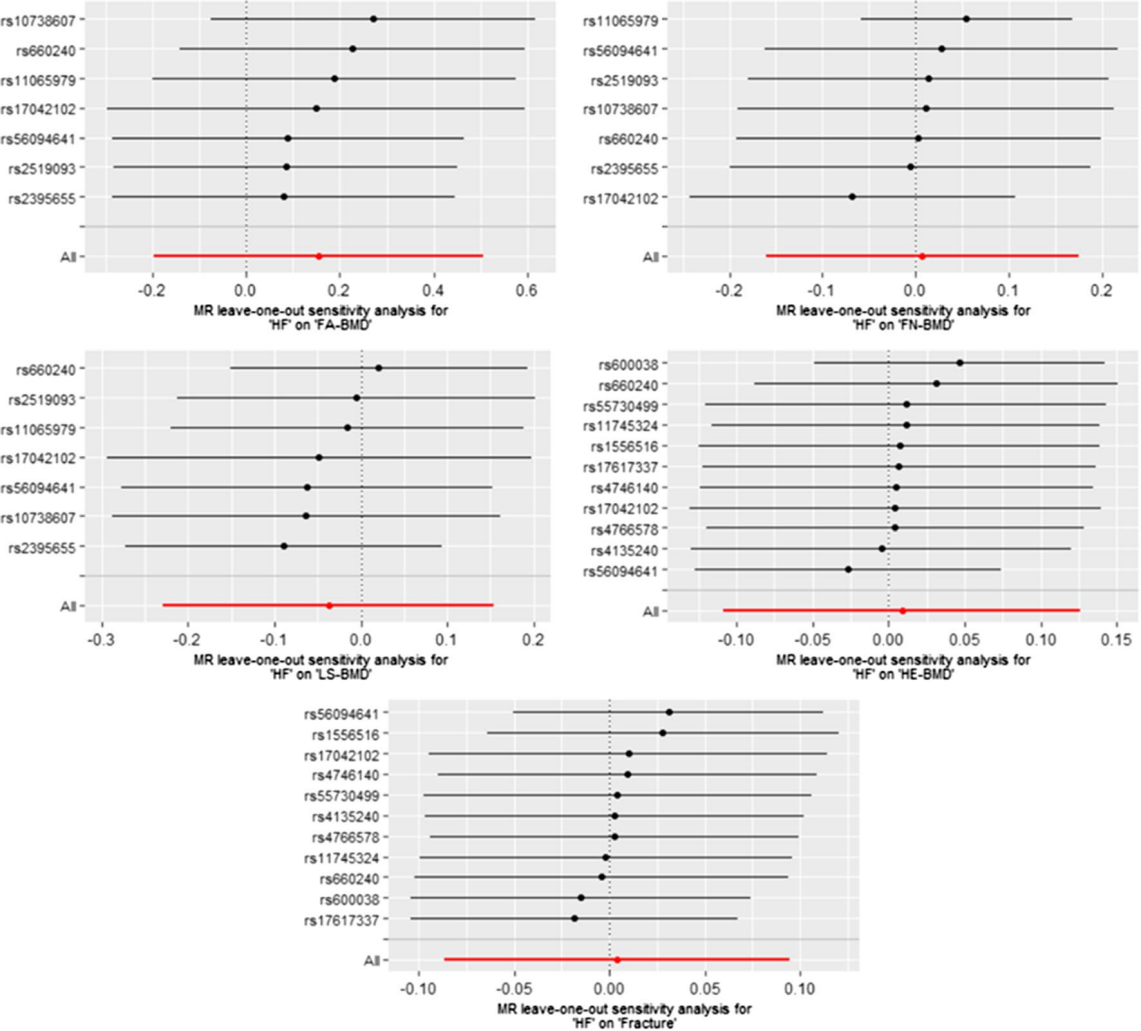
Leave-one-out plots for the MR analyses of HF on FA-BMD, FN-BMD, LS-BMD, HE-BMD, and fracture. HF, heart failure; FA-BMD, forearm bone mineral density; FN-BMD, femoral neck bone mineral density; LS-BMD, lumbar spine bone mineral density; HE-BMD, heel bone mineral density

Heterogeneity was detected in sensitivity analyses as suggested by Cochran’s Q and I^2^ statistics (Additional file 1: Table S3). However, the mre-IVW that we used can provide reliable estimates even in the presence of heterogeneity [33]. The MR-Egger intercept was close to zero (*P*_intercept_ > 0.05) for all considered outcomes except for FN-BMD (*P*_intercept_ = 0.033). No pleiotropic outliers were detected in the MR-PRESSO analysis (Additional file 1: Table S3). In addition, the MR-PRESSO method identified 4 outliers (rs4135240, rs56094641, rs56094641, and rs660240) for HE-BMD; and we observed that the null causal association persisted after excluding these outliers (OR 1.02; 95% CI 0.99, 1.05; *P* = 0.177; Table 2). Scatter plots describing the main results are shown in Fig. 3. The leave-one-out analyses showed no SNPs could potentially drive the null causal effects of HF on BMDs and fracture (Fig. 4).

## Discussion

We used summary statistics from different GWASs for the current two-sample MR study. The analyses provided little evidence that HF was causally associated with decreased BMD at different skeletal sites (including FA, FN, LS, and HE) or increased fracture risk.

Available data from observational studies over the past decade have suggested potential associations of HF with lower BMD and increased fracture risk. A meta-analysis revealed a significantly reduced BMD in patients with HF [36]. Similarly, another meta-analysis combining data from 7 cohort studies linked HF to an increased risk of fracture [37]. However, whether HF plays the role of driver or passenger in osteoporosis or fracture was largely unknown. No conclusion of causality can be drawn from the available data. The lack of causality in this MR study suggested that the association between HF and BMD and fracture observed in the observational studies may be biased by limited sample size, residual confounders, or misclassification. Our results corroborated a previous multicenter study suggesting the association between heart failure and hip fracture may be largely due to shared risk factors [38].

Aging is one of the most prominent confounders that influence both bone metabolism and cardiac function. More than 10% of older adults (70 + years) have HF [39]. Also, aging brings with it many changes in body composition and is considered a risk factor for a declined BMD [1]. As global aging progresses, both HF and osteoporosis represent major health threats. On the other hand, the rising prevalence of dementia places a heavy burden on the health care system [40]. Patients with dementia experienced higher hip fracture risk [41] and had a poor prognosis after hip fracture surgery [42]. Furthermore, prolonged use of loop diuretics in patients with HF is known for its effect on calcium homeostasis [43]. Increased plasma parathyroid hormone levels induced by loop diuretics treatment can mobilize calcium from cortical bone by enhancing turnover [44]. However, observational studies usually have difficulty controlling for these confounding factors [11, 12, 44]. Collectively, aging, dementia, and loop diuretics use that accompany heart failure, rather than heart failure itself, are likely to contribute to lower BMDs and increased risk of fracture that was observed in traditional studies.

MR methods for enhancing the reliability of the results included consensus methods (weighted median, simple median, and MR-egger method), outlier-robust methods (MR-PRESSO), modelling methods (MR-Egger intercept), and leave-one-out analysis. The no-causal results did not negate the previously observed associations of HF with BMD and fracture but provided greater insight into the mechanisms underlying these disorders.

Several strengths were notable in this study. First, the MR approach utilizing the largest GWAS to date diminished the residual confounders and inverse causality, which commonly occurs in traditional epidemiological studies. Second, several complementary analyses (Weighted median, simple median, MR-Egger regression, MR-PRESSO methods, and leave-one-out analysis) returned broadly consistent results, thus strengthening the causal inference. Third, the data sources that we used were confined to individuals of European ancestry. Therefore, our results are less susceptible to population structure bias.

There were some limitations worth noting. First, we did not explore the causality in the associations of HF with BMDs and fracture in other populations due to the lack of related GWAS datasets. This may limit the generalizability of our findings. Further studies on other populations are warranted. Second, despite the use of a set of sensitivity analyses, the bias introduced by horizontal pleiotropy and heterogeneity among the SNPs remains a concern. Finally, some of the fracture cases were identified by questionnaire-based self-reporting, which may introduce measurement bias.

## Conclusion

This two-sample MR study does not provide evidence of a causal association between HF and the risk of reduced FA-BMD, FN-BMD, LS-BMD, HE-BMD, or the increase risk of fracture in the European population.

## Supplementary Information

**Additional file 1:** **Table S1.** STROBE-MR Checklist [20]. **Table S2.** Genome-wide significant SNPs associated with HF and their association with BMDs and fracture. **Table S3.** Evaluation of heterogeneity and directional pleiotropy using different methods.

## Data Availability

The summary statistics of GWAS for heart failure are derived from a GWAS meta-analysis conducted by HERMES consortium (https://cvd.hugeamp.org/); Full GWAS summary statistics for FA-BMD, FN-BMD, and LS-BMD are publicly available through http://www.gefos.org/; Summary level data for HE-BMD and fracture can be accessed at https://www.ebi.ac.uk/gwas/downloads/summary-statistics.

## Abbreviations

HF: Heart failure
MR: Mendelian randomization
FA: Forearm
FN: Femoral neck
LS: Lumbar spine
HE: Heel
BMD: Bone mineral density
OR: Odds ratio
CI: Confidence interval
GWAS: Genome-wide association study
SNP: Single nucleotide polymorphism
IV: Instrumental instrument
IVW: Inverse variance weighted
mre: Multiplicative random effects
MR-PRESSO: MR-pleiotropy residual sum and outlier
